# Unravelling the effect of renal denervation on glucose homeostasis: more questions than answers?

**DOI:** 10.1007/s00592-023-02208-7

**Published:** 2023-12-08

**Authors:** Evaggelia Koutra, Kyriakos Dimitriadis, Nikolaos Pyrpyris, Panagiotis Iliakis, Christos Fragkoulis, Eirini Beneki, Alexandros Kasiakogias, Panagiotis Tsioufis, Fotis Tatakis, Athanasios Kordalis, Dimitrios Tsiachris, Konstantina Aggeli, Konstantinos Tsioufis

**Affiliations:** 1 First Department of Cardiology, School of Medicine, Hippokration General Hospital, National and Kapodistrian University of Athens, 115 27 Athens, Greece; 2 Dardanellion 146-148, 17123 Athens, Greece

**Keywords:** Renal denervation, Hypertension, Glycemic control, Autonomous nervous system, Insulin, Diabetes

## Abstract

Renal Denervation (RDN) is an interventional, endovascular procedure used for the management of hypertension. The procedure itself aims to ablate the renal sympathetic nerves and to interrupt the renal sympathetic nervous system overactivation, thus decreasing blood pressure (BP) levels and total sympathetic drive in the body. Recent favorable evidence for RDN resulted in the procedure being included in the recent European Guidelines for the management of Hypertension, while RDN is considered the third pillar, along with pharmacotherapy, for managing hypertension. Sympathetic overactivation, however, is associated with numerous other pathologies, including diabetes, metabolic syndrome and glycemic control, which are linked to adverse cardiovascular health and outcomes. Therefore, RDN, via ameliorating sympathetic response, could be also proven beneficial for maintaining an euglycemic status in patients with cardiovascular disease, alongside its BP-lowering effects. Several studies have aimed, over the years, to provide evidence regarding the pathophysiological effects of RDN in glucose homeostasis as well as investigate the potential clinical benefits of the procedure in glucose and insulin homeostasis. The purpose of this review is, thus, to analyze the pathophysiological links between the autonomous nervous system and glycemic control, as well as provide an overview of the available preclinical and clinical data regarding the effect of RDN in glycemic control.

## Introduction

Renal denervation (RDN) is an interventional procedure, which, by ablating the renal sympathetic nerves in close proximity to the renal arteries’ walls, ameliorates the renal sympathetic nervous system (SNS) interactions, that are largely responsible for the renal-induced sympathetic overdrive observed in pathologies including hypertension, heart failure (HF) and metabolic syndrome, and thereby results in reduction of arterial blood pressure (BP) [1–3]. The procedure itself has predominantly emerged as an adjunct to medication intervention for the management of hypertension and specifically resistant hypertension. Over the years, a large amount of randomized, sham controlled trials have proven the safety and efficacy of RDN in both short and long-term follow-up [4]. As a result, recent clinical practice consensuses from European and American Societies support the use of RDN in patients with resistant hypertension [5, 6], while the 2023 European Society of Hypertension Guidelines for the Management of Hypertension also include RDN as a third option for achieving BP targets in suitable patients with resistant hypertension [7]. These advances highlight the increasing role of RDN as a therapeutic tool in the arsenal of anti-hypertensive interventions, and is now considered by many the third pillar in the management of hypertension.

Glycemic control is an essential parameter of treating patients with hypertension, as well as other cardiovascular pathologies. Diabetes and cardiovascular disease are two of the most common comorbidities among adults, with more than 529 million people living with diabetes and 1.39 billion with hypertension worldwide [8, 9]. Also, diabetes is as an established risk factor for adverse health outcomes and increased cardiovascular mortality [10], while non-optimal glycemic control is also associated with a greater risk of developing hypertension [11]. Common pathophysiological links between hypertension and glucose homeostasis, and specifically the increased sympathetic overdrive evident in patients with hypertension and metabolism pathologies, emerge the idea of potentially managing to control both BP and glycemic status by ameliorating sympathetic overactivation, with the use of RDN.

The purpose of this review is, thus, to present the autonomous nervous system mechanisms which regulate glycemic status, the preclinical and clinical data regarding the effect of RDN in glucose control as well as the future perspectives of using RDN in patients with cardiovascular and metabolic pathologies.

## The pathophysiology of glycemic control via autonomous nervous system

### Autonomous nervous system and glycemic control

The sympathetic nerves are a part of the autonomic nervous system, regulating many functions of the human physiology. Central sympathetic neurons are located in the rostral ventrolateral medulla (RVLM), which is a key area for the regulation of BP and metabolism [12, 13]. The RVLM neurons signal directly the sympathetic preganglionic neurons, located in the spinal cord, and innervate several organs, while feedback is conveyed by a number of afferent inputs from carotid and organ receptors (i.e., mechanoreceptors, chemoreceptors), as well as hormones [14]. SNS plays an important role in regulation of daily energy expenditure by controlling metabolic rate, food intake and temperature. It has been generally recognized that increased sympathetic neural activity (SNA) results in catabolic effects on glucose and lipid metabolism, whereas increased parasympathetic neural activity results in anabolic effects. A number of afferent nerves from peripheral organs convey metabolic information that modulate activation of RVLM. Circulating molecules such as insulin and angiotensin, which are able to cross the blood–brain barrier can also influence central sympathetic outflow and thereby modulate peripheral lipid and glucose metabolism [15]. Moreover, sympathetic neurotransmitter norepinephrine and epinephrine are known to affect glucose transport and metabolism in the liver, pancreas, adipose tissue, and skeletal muscle. The liver, which plays a key role in glucose metabolism, is richly innervated by the autonomic components from the splanchnic sympathetic nerves and vagal parasympathetic nerves. Additionally, the part of sympathetic nerve fibers which innervate the liver arise directly from the hypothalamus, which is responsible for food intake and appetite regulation [16]. SNA and catecholamines increase glucose by activation of α1 and β2 receptors in the liver, leading to glycogenolysis and gluconeogenesis.

Furthermore, sympathetic nerves innervating skeletal muscle can modulate glucose uptake and glycogenolysis independent of concomitant increase in plasma insulin levels, via activation of β2 adrenergic receptors [17, 18]. Of note, administering a medical β2 agonist appears to improve glucose tolerance due to increased glucose uptake in skeletal muscle [19]. Conversely, neuronal stimulation of α-adrenergic receptors in arterioles elicits vasoconstriction, thus decreasing glucose utilization and leading to peripheral insulin resistance (IR). All these processes increase BP and glucose concentration in the blood, actions that are expected after activation of the SNS. However, SNS overactivation, due to chronic increase of stimulating factors or decreased activation of the parasympathetic system, contributes to the development of resistant hypertension, obesity, metabolic syndrome and IR.

### The role of kidneys in glucose homeostasis

The kidneys also play a role in glucose homeostasis by ensuring that glucose is not lost in urine through three membrane protein-sodium-glucose cotransporters (SGLT1, SGLT2, and GLUT2), which are responsible for glucose reabsorption [20]. Additionally, multiple studies have shown that the human kidney, through renal gluconeogenesis, releases significant amounts of glucose into circulation in the postprandial state under both physiological and pathological conditions and seems to be regulated by postprandial increases in SNS activity [21, 22].

**Fig. 1:**
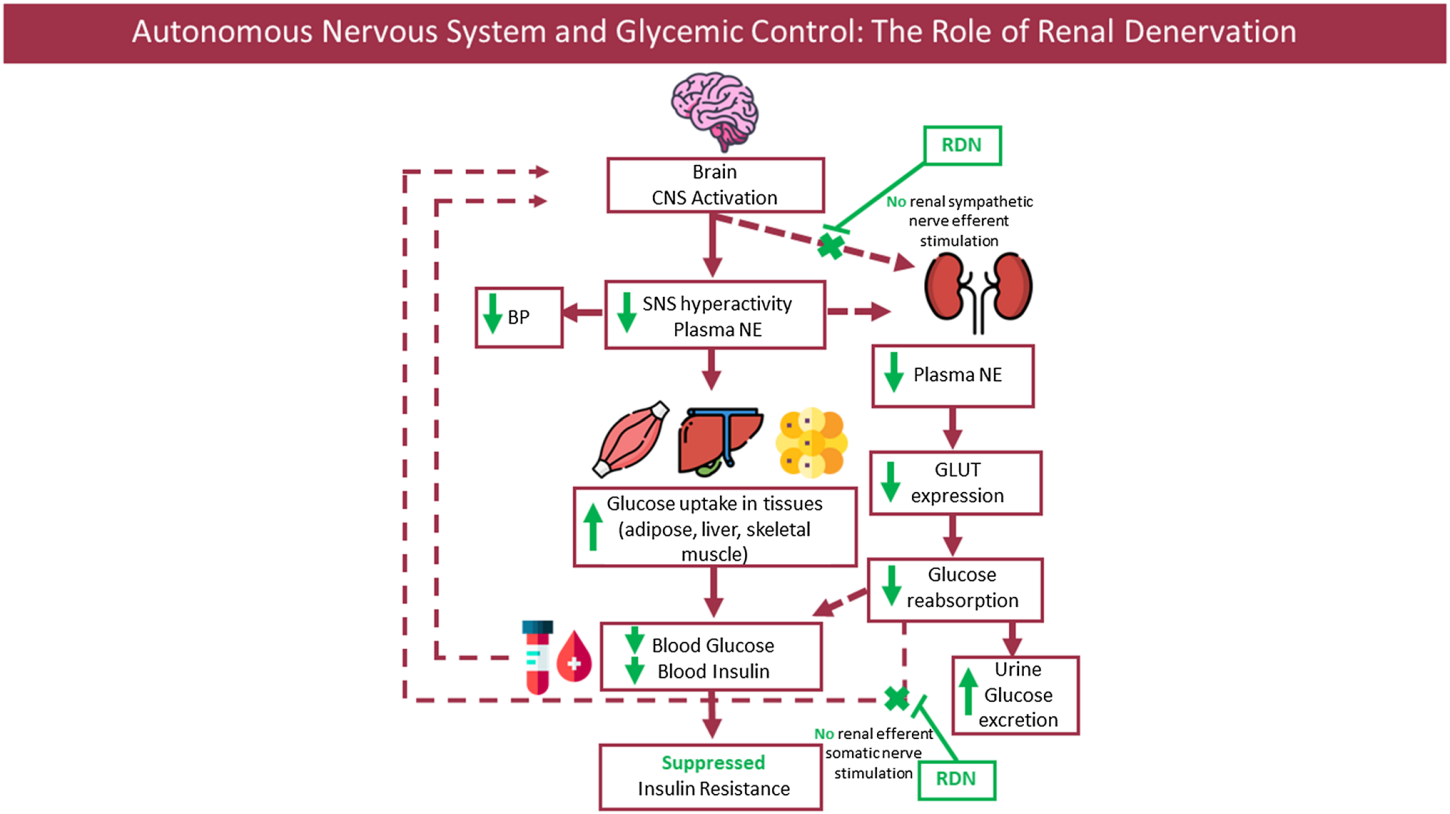
Schematic diagram summarizing the proposed mechanisms by which renal denervation modulates renal glucose metabolism. Abbreviations: CNS: Central Nervous System; SNS: Sympathetic Nervous System, NE: Norepinephrine; BP: Blood Pressure; RDN: Renal Denervation

Finally, intrarenal sympathetic hyperactivity may cause increased renal afferent nerve signaling, leading to further stimulation of the central SNS, thus initiating a vicious cycle (Fig. 1). RDN breaks this vicious cycle resulting in suppression of the SNS, and thereby improved glucose metabolism and insulin sensitivity. Therefore, there is a pathophysiologic interaction among insulin resistance, obesity and the central nervous system, which is potentially mediated by ameliorating renal sympathetic afferent and efferent signals. Interestingly, in the clinical use of a centrally acting sympatholytics drug, which are known to reduce central sympathetic activity [23], they reduce efferent sympathetic nerve traffic to the kidney, suppress glucose intolerance and increase skeletal muscle blood flow with less glycogenolysis. Therefore, an interventional procedure mimicking sympatholytics and reducing the renal-SNS crosstalk, such as RDN, may also lead to a benefit in the homeostasis of glucose and insulin, resulting in better disease control.

## Renal denervation and glycemic control: preclinical and clinical data

The importance of enhanced glycemic control in patients with hypertension and other cardiovascular diseases, as aforementioned, is essential. Despite the successful management of diabetic patients with antidiabetic medications, pharmacotherapy has several pitfalls, including polypharmacy and low adherence rates. Therefore, RDN, a one-time intervention, aiming to disrupt the pathophysiological link between diabetes and SNS overactivation in addition to achieving BP control, seems a promising therapeutic strategy. Several preclinical and clinical trials have investigated the effect of RDN in hypertension and concomitantly in the glycemic status of hypertensive patients, in order to determine any beneficial effect of the procedure in glucose and insulin metabolism.

### Preclinical data

One of the first animal trials that has studied the effect of denervation on glucose metabolism was in 1977, where Szalay et al. studied the effect of glucose per os loading on dogs that have been previously unilaterally surgically denervated [24]. They demonstrated that glucose tubular reabsorption in the kidney was significantly decreased in denervated dogs, compared to healthy controls.

Schaan et al. [25] evaluated the efficacy of bilateral denervation in rats with diabetic nephropathy; they studied 26 rats—13 of them with diabetic nephropathy and 13 healthy—that were randomized to either surgical RDN or no procedure, demonstrating a significant decrease in the levels of cortical GLUT1 protein toward normal values, without regression of diabetic-induced albuminuria. The same research team demonstrated, in an animal model of diabetic and non-diabetic rats, that there is direct association between renal sympathetic activity and GLUT2 levels, as denervation reduced GLUT2 levels in both non-diabetic and diabetic rats (-21% and -15%, respectively) and concluded that denervation modulates GLUT2 expression in kidney cells [26].

The multiple pleiotropic effects of surgical RDN on glucose metabolism were also demonstrated in an animal setting of uninephrectomised diabetic rats [27], in which denervation was associated with reduction in BP and renal tissue noradrenaline levels. RDN was also associated with improvement in glucose metabolism and insulin sensitivity, reflected by an increase in peripheral in-cell glucose uptake, as well as significant decrease of SGLT2 levels in renal cells, followed by attenuation of diabetic glycosuria.

As forewritten, insulin sensitivity plays a pivotal role in glucose metabolism and RDN could lead to attenuation of insulin sensitivity. Iyer et al. [28] carried out an animal study, showing that RDN could lead to regression of high hepatic insulin resistance. Specifically, they evaluated insulin sensitivity in a nonhypertensive obese canine model, before and after a 6-week period of high-fat diet, and after either surgical RDN or sham procedure. They reported a significant decrease of hepatic insulin sensitivity after RDN, suggesting a direct effect of RDN, via downregulation of hepatic gluconeogenesis through the natriuretic peptide pathway. Similar findings were demonstrated at another animal study by Chen et al. resulting that RDN, when applied unilaterally in high-fat-fed rats, led to 18.2% decrease of glucogenolysis and 16.3% decrease of gluconeogenesis; on the other hand, when applied bilaterally, it led to 31.9% decrease of glucogenolysis and 42.8% decrease of gluconeogenesis [29].

Another possible mechanism that denervation could lead to improved glucose tolerance and metabolism in wild-type and diabetic mice may be increased glycosuria, via the decreased expression of GLUT2 in renal tissue. These findings are supported by an animal study, in which improved glucose levels and increased glycosuria are met in mice undergoing renal denervation and in hypothalamic arcuate nucleus-specific pro-opiomelanocortin deficient (ArcPomc − / −) mice, demonstrating diminished renal sympathetic activity in both animal populations [30].

Although surgical denervation leads to glucose metabolism improvement, there were few data regarding the effect of catheter-based RDN. Pan et al. carried a study at which they divided 33 diabetic dogs into three arms: bilateral RDN arm, left RDN arm, and sham procedure arm [31]. They demonstrated that fasting plasma glucose (9.64 ± 1.57 mmol/L vs. 5.12 ± 1.08 mmol/L; *P* < 0.0001), fasting insulin (16.19 ± 1.43 mIU/mL vs. 5.07 ± 1.13 mIU/mL; *p* < 0.0001), and insulin resistance in the bilateral RDN arm, had statistically significant reduction, compared with the sham arm, supporting their hypothesis that multi-electrode catheter-based leads to significant reduction of gluconeogenesis and glycogenolysis 3 months post-intervention, without any procedure-related adverse events. Another animal study that evaluated the effect of increased sympathetic activity on hypertension and insulin sensitivity had similar findings [32]. The researchers included glucose-fed rats or fructose-fed rats that underwent bilateral RDN using cryoablation or sham denervation and demonstrated that RDN led to significant decrease of renal noradrenaline spillover and plasma renin activity, with an imminent effect not only in BP reduction but also to an improvement in glucose-to-insulin ratio and insulin sensitivity.

De Oiveira et al. also studied the effect of catheter RDN on diabetic mice [33]. For the purpose of the study, they divided mice into three groups: control, diabetic and diabetic that underwent RDN. They demonstrated that renal sympathetic activity was higher in diabetic rats, while RDN led to significant reduction of both glycemia and glycosuria, as well as reduction of SGLT2 expression on the kidney cells’ membrane and normalization of renal sympathetic activity. Regarding SGLT2 expression, newfangled evidence supports that SGLT2 expression is increased in the proximal tubule of the kidney, especially on the presence of HF [34]. In specific, the researchers performed RDN in rats with HF and demonstrated that sodium and glucose excretion increased, as a response to dapagliflozin. Moreover, in vitro high levels of noradrenaline induced translocation of SGLT2 to the cell membrane, highlighting the pleiotropic effects that RDN could have in human patients with HF, by not only reducing BP levels, but also improving glucose metabolism and altering the expression of SGLT transporters at renal tissue.

### Clinical data

Following the results of preclinical studies, a number of clinical trials investigating the use of RDN in hypertension also reported outcomes regarding glycemic control (Table 1). Bhatt et al. [35], in a randomized trial (SYMPLICITY HTN 3) assessing the safety and efficacy of RDN in patients with resistant hypertension, investigated the effect of RDN on the levels of Hba1C, along with outcomes regarding BP controls. The trial, which included 364 patients in the RDN arm and 171 in the sham procedure arm, reported no benefit of RDN in the levels of glycated hemoglobin in all patients, as well as in patients with diabetes. Similarly, Rosa et al. [36], in the Prague-15 study, which compared RDN to intensified pharmacotherapy with spironolactone in resistant hypertensives, showed no statistically significant reduction of fasting glucose levels in patients that received RDN treatment, compared to the pharmacotherapy group.

**Table 1:** Clinical Studies regarding the effect of renal denervation in parameters of glycemic control

Study	Year	Study type	Patient characteristics	Follow-up	Outcomes					
					Glucose	Insulin	C-Peptide	HbA1C	HOMA-IR	OGTT
Bhatt et al. [35]	2014	RCT	535 patients (364 RDN; 171 sham) with RHTN	6 months	Not reported	Not reported	Not reported	All patients: No significant difference (RDN: 0.06 ± 0.93%; Sham: − 0.06 ± 0.87%; *p* = 0.19), Diabetes patients: No significant difference(RDN: 0.12 ± 1.15%; Sham: − 0.22 ± 1.14%; *p* = 0.051)	Not reported	Not reported
Rosa et al. [36]	2015	RCT	106 patients with RHTN (52 RDN; 54 intensified pharmacotherapy)	6 months	Non-significant decrease of FGL, compared to control (RDN: − 0.5 (− 1.0, 0.02), *p* = 0.06 vs Pharmacotherapy: − 0.6 (− 1.2, 0.1) *p* = 0.07)	Not reported	not reported	Not reported	Not reported	Not reported
Bohm et al. [37]	2015	RCT	998 patients with RHTN	6 months	No significant difference: Diabetics: Baseline: 153.83 ± 62.71, 6 months: 138.91 ± 48.29; *p* < 0.05; Non-diabetics: Baseline: 107.51 ± 111.26, 6 months: 101.88 ± 26.29; *p* < 0.05)	Not reported	Not reported	Not reported	Not reported	Not reported
Pourmoghaddas et al. [38]	2016	Prospective	30 patients with RHTN	1 year	Significant reduction of FGL compared to baseline (111.7 ± 15.7 vs. 107.2 ± 12.9; *p* = 0.001)	Not reported	Not reported	Not reported	Not reported	Not reported
Hopper et al. [39]	2017	Observational, Prospective	39 patients with RHTN and renal failure	1 year	Not reported	Not reported	Not reported	Not reported	Not reported	Significant reduction of the 120-OGTT(11.2 ± 5.1 vs 9.9 ± 3.6; *p* = 0.026)
Aripov et al. [40]	2017	Observational, Retrospective	63 patients with RHTN	1 year	Not reported	Significant reduction in HOMA-IR (3.0 ± 4.6 vs. 2.5 ± 3.7, *p* = 0.007)	Not reported	Not reported	Not reported	Not reported
Mahfoud et al. [41]	2011	Controlled Clinical Trial	50 patients (37 RDN; 13 control)	3 months	FGL in RDN group: From 118 ± 3.4 to 108 ± 3.8 mg/dL (*P* = 0.039) Control didn’t had any significant changes	After RDN, insulin levels were significantly decreased from 20.8 ± 3.0 to 9.3 ± 2.5 μIU/mL (*p* = 0.006) Control didn’t had any significant changes	In RDN group: Significant reduction (5.3 ± 0.6 to 3.0 ± 0.9 ng/mL; *p* = 0.002) Control didn’t had any significant changes	Not reported	Significantly decreased HOMA-IR index (from 6.0 ± 0.9 to 2.4 ± 0.8; *p* = 0.001) in the RDN group	2 h OGTT was reduced by 27 mg/dL (*p* = 0.012) in the RDN arm
Matous et al. [42]	2015	Observational, Retrospective	51 patients with RHTN	12 months	FGL was significantly increased (baseline: 7.4 ± 2.0 mmol/L; 1 year: 7.8 ± 2.6 mmol/L; *p* = 0.032)	Not reported	No difference in C-peptide levels (baseline: 1178 ± 429 pmol/L; 1 year: 1271 ± 565 pmol/L; *p* = 0.098)	Post-RDN, higher, but not significantly, levels of HbA1c (baseline: 46.1 ± 10.5 mmol/mol; 1 year: 47.6 ± 13.6 mmol/mol; *p* = 0.079)	Not reported	Not reported
Verloop et al. [43]	2015	RCT	29 patients with metabolic syndrome and indication for RDN	6 months and 12 months	No significant difference in FGL (baseline: 7.2 ± 1.7 mmol/L; 6 months: 7.4 ± 2.6 mmol/L; 12 months: 7.0 ± 1.3 mmol/L; *p* = 0.34 for both)	No significant difference in FIL (baseline: 20.9 ± 10.6 mIU/L; 6 months: 20.1 ± 9.8 mIU/L; 12 months: 19.6 ± 11.1 mIU/L; *p* = 0.53 for both) Insulin sensitivity was not different at 6 months (median change, 0.00; *p* = 0.60) and 12 months after RDN (median change, − 0.001; *p* = 0.77)	No significant difference in C-peptide levels (baseline: 1319 ± 410 pmol/L; 12 months: 1306 ± 468 pmol/L; *p* = 0.82)	Not reported	Not reported	Not reported
Tsioufis et al. [44]	2017	RCT	17 patients with metabolic syndrome undergoing RDN	3 months	Not reported	Not reported	Not reported	Not reported	No significant change in HOMA-IR	Improved sympathetic response to OGTT (30 min OGTT: burst frequency increased to 52 ± 8 bursts per minute (p < 0.001;120 min: burst frequency increased to 54 ± 8 bursts per minute (*p* = 0.004)
Miroslawska et al. [45]	2016	Observational, Prospective	18 patients with metabolic syndrome	4 months	No significant changes	No significant changes in insulin levels or insulin sensitivity	No significant changes	Not reported	Not reported	Not reported
Kampmann et al. [46]	2017	Observational, Open label	8 patients with RHTN	6 months	Endogenous glucose production, was non-significantly decreased during both the basal and clamp period (1.73 ± 0.16 versus 1.36 ± 0.19 mg/kg/min; *p* = 0.27 and 0.62 ± 0.14 versus 0.36 ± 0.28 mg/kg/min; *p* = 0.52, respectively)	No significant changes in insulin levels Insulin sensitivity was non-significantly improved (baseline M-value: 2.68 ± 0.28 mg/kg/min; 6 months; 3.07 ± 0.41 mg/kg/min; *p* = 0.12)	No significant changed	Not reported	Not reported	Not reported
Eikelis et al. [47]	2017	Observational	57 patients with RHTN	3 months	Not reported	FIL was significantly higher after RDN (baseline: 20.05 ± 1.46 Uu/mL; 3 months: 29.70 ± 2.51 uU/ml; *p* = 0.002)	Not reported	Not reported	Not reported	Not reported
Witkowski et al. [48]	2011	Observational	10 patients with SA and RHTN	6 months	Not reported	Not reported	Not reported	Significant decrease (median baseline: 6.1%; median 6 months: 5.6%; *p* < 0.05)	Not reported	Significant decrease of glucose at 120 min OGTT (median baseline: 7.0 mmol/L; median 6 months: 6.4 mmol/L; *p* = 0.05)
Daniels et al. [49]	2017	Observational	20 patients with SA and RHTN	6 months	FGL: non-statistically significant reduction (−0.22 mmol/L, 95% CI: −0.22, −0.77; *p* = 0.46)	Not significantly changed	Not reported	Not significantly changed	Not reported	Glucose levels were significantly reduced after 120 min OGTT (−1.14 mmol/L; 95%CI: −0.22, −2.06; *p* = 0.03)
Warchol-Celinska et al. [50]	2018	Prospective Randomized	60 patients with SA and RHTN (30 RDN; 30 control)	3 months	FGL: No significant difference in the RDN arm (baseline: 6.8 ± 2.1 mmol/L; 3 months: 7.1 ± 2.9 mmol/L; *p* = 0.70)	FIL: No significant difference in the RDN arm (baseline: 13.4 ± 9.3 mmol/L; 3 months: 12.8 ± 9.8 mmol/L; *p* = 0.48)	Not reported	No significant difference after RDN (baseline: 6.3 ± 1.1%; 3 months: 6.5 ± 1.8%; *p* = 0.79)	Not reported	Not reported
Manukyan et al. [51]	2022	Observational, Prospective	59 patients with type 2 DM and RHTN	1 year	FGL: No significant change in patients with RRI ≥ 0.7 (+ 0.12 mmol/L; 95% CI: − 0.92, 1.17; *p* = 0.804) and RRI < 0.7 (+ 0.22 mmol/L; 95% CI: − 0.70,0.25; *p* = 0.337)	Not reported	Not reported	No significant change in patients with RRI ≥ 0.7 (+ 0.11%; 95% CI: − 0.41, 0.64; *p* = 0.660) and RRI < 0.7 (− 0.13%; 95% CI: − 0.80,0.53; *p* = 0.674)	Not reported	Not reported
Miroslawska et al. [52]	2021	Observational	20 non-diabetic patients with RHTN	24 months	FGL: No significant changes observed (baseline: 5.9 ± 0.8 mmol/L; 24 months: 5.7 ± 0.9 mmol/L; *p* = 0.08)	FIL: No significant changes observed (baseline: 134 ± 85 pmol/L; 24 months: 159 ± 54 pmol/L; *p* = 0.29)	Fasting C-peptide levels not significantly different (baseline: 1242 pmol/L, 95% CI: 890–2509; 24 months: 1477 pmol/L, 95%CI: 1002–2295; *p* = 0.13)	HbA1C not significantly changed (baseline: 5.6 ± 0.3%; 24 months: 5.6 ± 0.5%; *p* = 0.93)	HOMA-IR not significantly changed IR (baseline: 6.3 ± 3.9; 24 months: 7,0 ± 2.9; *p* = 0.45)	Not Reported

*HTN* hypertension, *RHTN* resistant hypertension, *DM* diabetes mellitus, *HOMA-IR* homeostasis model assessment-insulin resistance, *OGTT* oral glucose tolerance test, *FGL* fasting glucose level, *FIL* fasting insulin level, *IS* insulin sensitivity, *SA* sleep apnea; *95% CI* 95% Confidence interval, *RRI* renal resistance index

Following studies, however, also investigating the role of RDN in BP control, showed benefit after RDN in several parameters of glucose control. In specific, Bohm et al. [37] evaluated the RDN-associated BP reduction in patients with hypertension, as well as the HbA1c and fasting glucose levels. The study, which enrolled a total of 998 patients, showed no difference between baseline and 6 months levels of HbA1c in both diabetics and non-diabetics, while also reporting significant reduction of fasting glucose levels in both aforementioned groups (*p* < 0.05). Similar reduction in fasting glucose levels were observed in a study by Pourmoghaddas et al. [38], which included 30 patients with resistant hypertension undergoing RDN. The investigators reported a reduction in fasting glucose levels of −4.50 mg/dL at 1-year follow-up, as well as significant reduction in BMI and waist circumference, parameters associated with metabolic syndrome (*p* = 0.008 and *p* = 0.003, respectively).

Moreover, Hopper et al. [39], in the SYMPLICITY HF Feasibility study, which included 39 patients with HF and renal impairment, showed significant reduction of the 120-min oral glucose tolerance test (OGTT) at 12 months, compared to baseline. Also, similar studies have shown possible alterations of insulin sensitivity mediated by RDN. More specifically, Aripov et al. [40], in a study assessing RDN’s effect in 63 patients with resistant hypertension, noted a significant reduction of the homeostasis model assessment-insulin resistance (HOMA-IR) index, which was significantly reduced by a mean of 0.5 at 12 months.

Mahfoud et al. [41], in a pilot study, aimed to assess the impact of RDN in several aspects regarding glucose and insulin homeostasis. They enrolled 50 patients, out of which 37 underwent RDN and 17 served as control, and they evaluated them 1- and 3-month post-intervention. Besides significant reductions in BP in both follow-up periods, the study also revealed significant decrease in fasting glucose levels, insulin and C-peptide levels in the RDN cohort. Furthermore, patients undergoing RDN had significantly decreased HOMA-IR index and 2 h OGTT was reduced by 27 mg/dL (*p* = 0.012). Interestingly, similar changed were not observed in the control group, implying the positive effect of RDN in glycemic control markers.

Despite earlier positive results, Matous et al. [42], in a subsequent study, enrolled 51 resistant hypertensives, which were grouped regarding the presence of diabetes and the number of radiofrequency applications to each renal artery (greater or lower than 4). Regardless of the group on which each patient was assigned, the investigators reported significantly increased fasting levels of glucose, higher levels of HbA1c and no difference in the levels of C-peptide.

Similarly, Verloop et al. [43], in the DREAMS randomized controlled trial, evaluated the effect of RDN in patients with metabolic syndrome. In particular, they enrolled 29 patients with metabolic syndrome undergoing RDN and assessed, besides BP post-intervention, a number of metabolic markers and insulin sensitivity. The median insulin sensitivity, as assessed by the simple index assessing insulin sensitivity OGTT (SIiSOGTT) was not different at 6 months and 12 months after RDN. Furthermore, fasting glucose levels, fasting insulin levels and C-peptide levels were not significantly changed post-RDN.

Tsioufis et al. [44] also investigated the effect of RDN in the sympathetic control of glycemic status. In specific, the enrolled 17 hypertensive patients which fulfilled 4 or more criteria for metabolic syndrome, and assessed muscle sympathetic nerve activity (MSNA) both at rest and during 75 g OGTT. At 3-month follow-up, the investigators reported a reduction of MSNA bursts at rest and improved sympathetic response to OGTT in the RDN group. However, no significant improvement was noticed in the HOMA-IR index (p = NS).

In another study, Miroslawska et al. [45] investigated insulin resistance in 23 patients with resistant hypertension, undergoing RDN, using a hyperinsulinemic-euglycemic step clamp (HEC). Out of them, 18 met the criteria for metabolic syndrome. At 4 months after RDN, mean fasting plasma glucose, median insulin and C-peptide concentration remained unchanged, compared to baseline. Furthermore, during HEC, the endogenous glucose production remained unchanged, while at high-dose insulin infusion, the measured glucose disposal remained unchanged to baseline. These results, thus, were indicative of no effect of RDN in peripheral and hepatic insulin sensitivity in patients with resistant hypertension.

Kampmann et al. [46], in their respective trial, aimed to study the effect of RDN in patients with resistant hypertension without diabetes. They included, therefore, 8 patients undergoing RDN, which were re-examined regarding BP metabolic parameters at 6 months. Regarding insulin sensitivity, as assessed by HEC and expressed as M-value, it was improved non-significantly, however investigators reported a significant correlation between greater insulin improvement and lower BMI (*R* = 0.75, *p* = 0.03). Moreover, the endogenous glucose production, as assessed by a 3-^3^H glucose tracer, was also non-significantly decreased during both the basal and clamp period of the test. Finally, no difference was reported in insulin, C-peptide, IGF-1 and glucagon levels.

Eikelis et al. [47], in a trial investigating how RDN affects the adipokine profile of patients with resistant hypertension, also investigated the response of insulin levels to sympathetic denervation. The study, which included 57 patients, showed at 3-month follow-up, besides a significant increase in adiponectin concentration, a significantly higher fasting insulin concentration after RDN, suggesting a possible positive effect in both lipid and glucose homeostasis.

Witkowksi et al. [48], in a trial assessing RDN in patients with resistant hypertension and sleep apnea, enrolled 10 patients, which were also followed up for glycemic control markers. The study showed that, in resistant hypertensive patients with sleep apnea, there was a significant decrease of plasma glucose concentration 2 h post-glucose administration, as well as in HbA1c levels.

Similarly, Daniels et al. [49] also investigated the effect of RDN in 20 patients with RDN and sleep apnea. At 6-month follow-up, the investigators reported a non-statistically significant reduction of fasting glucose levels and a significant reduction of glucose levels following a 120 min OGTT. Other parameters, such as insulin sensitivity, HbA1c and percentage of pancreatic beta cell function, did not alter significantly from baseline at the time of the follow-up. Moreover, another study by Warchol-Celinska et al. [50] involving 60 patients with resistant hypertension and sleep apnea, also examined the effect of RDN on glycemic control variables. The total cohort of patients was randomly assigned to either RDN arm (*n* = 30) or control arm (*n* = 30). Regarding glucose metabolism, at 3 months the study did not show any change from baseline at the RDN arm in fasting glucose levels, fasting insulin levels or HbA1c levels.

A following study by Manukyan et al. [51], which aimed to evaluate RDN and its relation to renal resistance and function, also evaluated several parameters in regards to glycemic control. The trial included 59 patients with resistant hypertension and type 2 diabetes. At 12-month follow-up, the investigators reported no significant changes in fasting plasma glucose and HbA1c, in both patients with a renal resistance index (RRI) ≥ 0.7 and < 0.7.

Finally, an observational study by Miroslawska et al. [52] evaluated long-term metabolic effects of RDN in patients with resistant hypertension. In specific, 20 resistant hypertensives without diabetes underwent RDN and were followed up at 6 and 24 months. The study reported no significant change in several metabolic parameters, such as HbA1c, fasting glucose levels, fasting insulin levels, fasting C-peptide levels and HOMA-IR.

## Clinical perspectives

As thoroughly described thus far, preclinical data suggest that RDN, both surgical and transcatheter, modulate glycose homeostasis by altering the expression of several renal receptors mediating glucose transfer, thus resulting in enhanced glycemic control. However, there is more uncertainty regarding evidence from clinical trials. In specific, even though there are a number of clinical trials investigating the effect of RDN in several parameters of glycemic status, the reported outcomes are conflicting, with several trials documenting a benefit of RDN in metabolic parameters and others finding no significant difference, compared to baseline. Nevertheless, a recent meta-analysis [53] including 19 studies and 2245 patients, showed no significant difference after RDN in fasting glucose levels (weighted mean difference [WMD]: 0.19 mmol/L; 95% CI: 0.37, 0.00 mmol/L), insulin levels (standardized mean difference [SMD]: 0.01; 95% CI: 0.41, 0.39), C-peptide levels (SMD: 0.05; 95% CI—0.30, 0.21), HbA1C levels (SMD: 0.05; 95% CI: 0.17, 0.07) or HOMA-IR (SMD: 0.29; 95% CI: 0.72, 0.14). Therefore, the available, to date, data suggest a modest or no effect of RDN in glycemic control, while the inconsistent results among clinical trials necessitate further research in the field.

Besides the importance of maintaining euglycemia in hypertension, glucose control is also important in other cardiovascular pathologies, including HF and atrial fibrillation (AF). In fact, regarding HF, it is established that diabetes can predispose to HF [54], while poor glycemic control may also contribute to deterioration of left ventricular function [55]. In respect to AF, the presence of diabetes, along with AF, seems to be connected with increased stroke rates [56], as well as increased all-cause and cardiovascular mortality and morbidity rates [57]. RDN has recently shown positive effects on the management of both HF [58–60] and AF [61–63], as both conditions are influenced by SNS overactivation. However, the effect of RDN in glucose control in such patients has not been yet investigated. Future research should, therefore, further investigate a potential benefit of RDN in both pathologies, especially in association with parameters of glycemic control.

Finally, it is of note that a large number of trials were not specifically designed to assess glycemic control, rather than metabolic parameters were reported as a result of blood tests in patients undergoing RDN for resistant hypertension. Consequently, there is no regard in the majority of trials for antidiabetic medications or lifestyle changes, which may have impacted patients’ glycemic status during the course of the study. Furthermore, the tested RDN methods mostly include radiofrequency ablation and not other modalities, such as ultrasound and alcohol RDN. Therefore, there are still several gaps and limitations, and more research is needed, in order to fully understand the possible role of RDN in maintaining glycemic controls in individuals with cardiovascular disease. Thus, future research should also focus in designing specific randomized, controlled RDN trials, powered to assess the impact of RDN in glucose homeostasis, as well as properly assess the effect of other RDN delivery modalities and the effect on glycemic status in patients with other cardiovascular pathologies.

## Conclusion

Despite preclinical data showing a benefit of RDN in maintaining glycemic control via several molecular mechanisms, available evidence from clinical studies are conflicting and do not unquestionably imply a clinical improvement in parameters related with glucose and insulin homeostasis. It is, therefore, certain that more research is needed in this field, in order to better understand the role of both sympathetic overdrive and RDN in glycemic control. Finally, future trials should also focus in patients’ populations besides hypertensives, such as individuals HF and AF, in order to investigate a potential effect of RDN in glycemic control in these pathologies.
